# Exploratory study of the impact of perceived reward on habit formation

**DOI:** 10.1186/s40359-018-0270-z

**Published:** 2018-12-20

**Authors:** Gaby Judah, Benjamin Gardner, Michael G. Kenward, Bianca DeStavola, Robert Aunger

**Affiliations:** 1 Department of Surgery and Cancer, Imperial College London, QEQM Building, St Mary’s Hospital Campus, Praed Street, London, W2 1NY UK; 2 Department of Psychology, Institute of Psychiatry, Psychology and Neuroscience (IoPPN), King’s College London, AH2.12, Addison House, Guy’s Campus, London, SE1 1UL UK; 3 London School of Hygiene and Tropical Medicine, Ashkirk, Scotland; 4 UCL Great Ormond Street Institute of Child Health, 30 Guildford Street, London, WC1N 1EH UK; 5 Department of Disease Control, London School of Hygiene and Tropical Medicine, Keppel Street, London, WC1E 7HT UK

**Keywords:** Automaticity, Habit formation, Behaviour change, Reward, Intervention

## Abstract

**Background:**

Habits (learned automatic responses to contextual cues) are considered important in sustaining health behaviour change. While habit formation is promoted by repeating behaviour in a stable context, little is known about what other variables may contribute, and whether there are variables which may accelerate the habit formation process. The aim of this study was to explore variables relating to the perceived reward value of behaviour – pleasure, perceived utility, perceived benefits, and intrinsic motivation. The paper tests whether reward has an impact on habit formation which is mediated by behavioural repetition, and whether reward moderates the relationship between repetition and habit formation.

**Methods:**

Habit formation for flossing and vitamin C tablet adherence was investigated in the general public following an intervention, using a longitudinal, single-group design. Of a total sample of 118 participants, 80 received an online vitamin C intervention at baseline, and all 118 received a face-to-face flossing intervention four weeks later. Behaviour, habit, intention, context stability (whether the behaviour was conducted in the same place and point in routine every time), and reward variables were self-reported every four weeks, for sixteen weeks. Structured equation modelling was used to model reward-related variables as predictors of intention, repetition, and habit, and as moderators of the repetition-habit relationship.

**Results:**

Habit strength and behaviour increased for both target behaviours. Intrinsic motivation and pleasure moderated the relationship between behavioural repetition and habit. Neither perceived utility nor perceived benefits predicted behaviour nor interacted with repetition. Limited support was obtained for the mediation hypothesis. Strong intentions unexpectedly weakened the repetition-habit relationship. Context stability mediated and for vitamin C, also moderated the repetition-habit relationship.

**Conclusions:**

Pleasure and intrinsic motivation can aid habit formation through promoting greater increase in habit strength per behaviour repetition. Perceived reward can therefore reinforce habits, beyond the impact of reward upon repetition. Habit-formation interventions may be most successful where target behaviours are pleasurable or intrinsically valued.

**Electronic supplementary material:**

The online version of this article (10.1186/s40359-018-0270-z) contains supplementary material, which is available to authorized users.

## Background

Habitual behaviours are those automatically elicited by environmental cues, due to the activation of mental cue-behaviour associations, which strengthen through repeated performance in a consistent context [1, 2]. Habits are advocated as a means to sustained behaviour change, due to their key feature of being automatically prompted by contextual cues, rather than relying on conscious input, memory or strong motivation [3, 4]. Automaticity is thought to be the ‘active ingredient’ of habit; it is because habitual actions are automatic that habit strength predicts and sustains behaviour, and thus why researchers are interested in habit formation as a means to establish healthy behaviours. Therefore, automaticity is often measured as an indicator of habit [5]. Greater understanding of the habit formation process is of theoretical importance, and understanding how behaviours become automatic may inform the design of interventions to support sustained behaviour change.

A study modelling habit formation over time for healthy eating, drinking and exercising behaviours (by measuring automaticity) found considerable variation in the time taken for habit to plateau (from 18 to 254 days) [5], indicating considerable variation between individuals and behaviours in the time taken to form a habit. While some studies have investigated correlates of habit strength [6, 7], there has been little experimental investigation of predictors of habit *formation* to date [8].

Promoting habit formation requires forming intentions for a new behaviour, translating intentions into action, sustaining action and repeated performance in specific contexts [9]. One factor cited as a potential predictor of habit formation is reward, which may play multiple roles in the habit formation process. Within social and health psychology, most accounts of habit formation describe the impact of reward on habit formation as being mediated by increased levels of repetition [5, 10]; (but see [11]) such that more rewarding behaviours are subsequently more frequently performed. Indeed, satisfaction with the outcomes of behaviour (one of many potential reward indicators) has been proposed to affect repetition through decisional processes, such as intention. For instance, more satisfying – and so rewarding – outcomes increase intentions to subsequently repeat behaviour [3]. This suggests that the effect of perceived reward on habit formation may be *mediated* by increased frequency of behaviour, and perhaps also by intentions.

However, the animal learning and neuroscience literature suggests that reward may *moderate* the impact of repetition on habit formation, by strengthening stimulus-response (i.e. context-behaviour) associations that underlie habit [12, 13]. That is, with the same number of repetitions, a rewarded behaviour may become habitual more quickly than an unrewarded behaviour*.* A recent lab study showed that concern for health led to greater formation of habitual healthy food choices [14], possibly because health-concerned participants found the healthy choice more rewarding. Other studies [6, 7] also indicate that perceived rewards may strengthen the impact of repetition on habit. These studies modelled variation in the strength of existing, stable habits, however studying predictors of habit formation requires tracking development of new habits over time.

The present study used a longitudinal design to explore whether the impact of rewards on habit formation is mediated by behavioural repetition, or rewards moderate the relationship between repetition and habit formation, or whether rewards act as both mediator and moderator. Better understanding of the psychological process of habit formation could identify ways to accelerate gains in habit strength, which should in turn sustain behaviour change [3].

### Potential reward indicators

Multiple psychological variables were investigated in the present study, as potential indices of reward. Pleasure is defined as a positive and immediate sensory outcome, similar to the food or drug rewards commonly used in the animal literature [15]. Physical pleasure may thus serve as a reward in human habit formation.

Intrinsic motivation (being motivated to act due to the anticipated inherent enjoyment of doing so) is more likely to lead to stronger intentions and sustain changes in behaviour than extrinsic motivation (being motivated to act to achieve a desired outcome of the behaviour, e.g. pleasing others) [16]. Observational studies of existing fruit consumption and exercise habits found that greater intrinsic motivation was associated with stronger habits, and reinforced the relationship between behavioural repetition and habit [6, 7].

Positive outcome expectancies have been found to be associated with formation of stronger habits, independent of behaviour repetition [11]. Positive evaluation of outcomes predicts behaviour maintenance [17]. Thus, performing behaviours believed to yield positive outcomes may be rewarding. Perceived positive outcomes could either be general beliefs about whether or not a behaviour is beneficial (perceived utility), or measures of specific outcomes expected from a particular behaviour (perceived benefits).

Two target health behaviours (flossing, vitamin C adherence) were investigated as they are: relatively mechanically simple (i.e. not comprising multiple sub-components requiring sustained attention); should be frequently performed (i.e. once a day); and can be performed in a constant context, so could feasibly become habitual [9]. Flossing removes plaque from areas that brushing does not reach, preventing cavities and gum disease [18]. Flossing is most effective when performed daily [19], and is typically performed in an unvarying context (the bathroom). Flossing has been previously studied in work on habits and habit formation [1, 11, 20]. Taking vitamin C tablets is also a simple behaviour that warrants once-daily performance, and which can be promoted given simple interventions [21].

### Hypotheses

1. The following psychological variables will function as rewards, through positively affecting the habit formation process:
  - pleasure,
  - intrinsic motivation,
  - positive outcome expectancies (in terms of perceived utility and perceived benefits).
2. The rewarding variables above will affect habit (measured using automaticity) via the following mechanisms:
  - The positive effect of reward on automaticity will be *mediated* by increased behaviour repetition (i.e. reward affects automaticity gain through promoting increased behaviour repetition).
  - reward will *moderate* the relationship between repetition and automaticity, such that stronger rewards will lead to greater increases in automaticity when repetition frequency is held constant.

## Methods

### Participants

Participants (*N* = 118, M_age_ = 35.7 years, SD = 11.8; 53 men, 65 women), living in London, UK, were members of the general public recruited by a market-research recruitment company. All participants received a flossing intervention, and the final 80 participants recruited received a vitamin C tablet intervention^1^ (M_age_ = 35.1 years, SD = 11.8). The study was explained verbally and in a written information sheet. All participants provided informed, written consent.

To facilitate the investigation of habit formation rather than bolstering existing habits, inclusion criteria were: typically floss no more than twelve times a month (i.e. three times a week) at recruitment; ‘sometimes’, ‘rarely’ or ‘never’ take vitamin C tablets; ‘definitely’, ‘probably’, or ‘maybe’ willing to try to floss and take vitamin C tablets more frequently.

### Design and procedure

This intervention study used a longitidinal single-group design. Behavioural repetition, habit and all self-report measures were recorded via online questionnaires every four weeks for a 16 week period, resulting in five timepoints (T0-T4). The vitamin C intervention took place at baseline (T0), and the flossing intervention four weeks post-baseline (T1). The interventions were at different timepoints to avoid competition between behaviours at the initiation phase, due to potential self-control or memory limitations [22]. As the study was investigating the impact of (unmodified) covariates on the habit formation process, a control group was unnecessary, therefore all participants received the habit formation intervention. There were home visits at T0, T1 and T4. The study procedure is outlined in Table 1. The study received institutional ethical approval.

**Table 1:** Study procedure

Timepoint	Home visit focus	Online questionnaire content
T0: 0 weeks (Baseline)	Consent Given floss and vitamin C tablets	*Online vitamin C intervention* Measures: Behaviour self-report, automaticity, context stability, intention, rewarding variables
T1: 4 weeks	*Face-to-face flossing intervention*	Measures: Behaviour self-report, automaticity, context stability, intention, rewarding variables
T2: 8 weeks	n/a	Measures: Behaviour self-report, automaticity, context stability, intention, rewarding variables
T3: 12 weeks	n/a	Measures: Behaviour self-report, automaticity, context stability, intention, rewarding variables
T4: 16 weeks	Semi-structured interview	Measures: Behaviour self-report, automaticity, context stability, intention, rewarding variables

Note: Due to data collection problems, the context stability items were not tested for the first 38 participants

### Interventions

Participants were provided with floss and vitamin C tablets at T0. The intervention techniques are specified according to the Behaviour Change Techniques Taxonomy v1 [23].

#### Vitamin C intervention

The online vitamin C intervention was delivered at T0, embedded within the study questionnaire. Information was presented about the function of vitamin C, and possible benefits of vitamin C supplements (‘information on health consequences’). To encourage engagement with intervention materials, participants were asked the extent to which they think they could achieve each benefit through taking vitamin C tablets. (These responses formed the ‘perceived benefits’ variable.) Participants were instructed to record precisely when in their routines they would take vitamin C tablets (‘implementation intentions’).

In order to boost the intervention, within the T1 questionnaire participants were asked three multiple choice questions about benefits of vitamin C, then given correct answers and explanations (‘health consequences’). Also at T1, participants were asked when they take their vitamin C tablet, whether this was a good time for them to take it, and whether they wanted to try taking it at a more convenient time (‘coping planning’, ‘reviewing behavioural goals’). They were asked if they forgot to take the tablet because they could not see it, and whether they wanted to move it to a more visible place (‘restructuring physical environment’).

#### Flossing intervention

This occured at T1, in an individual session with the researcher, lasting 30–40 min. Participants were given an information leaflet, (also explained orally by the researcher) describing positive ‘health consequences’ and ‘social consequences’ of flossing, and instructions on how and when to floss.^2^ Participants were guided in forming ‘implementation intentions’ and specified when and where to floss [24], based on their own personal routines. This was written on the leaflet, and read aloud, along with a pledge to floss every night, to establish ‘commitment’ and a ‘behavioural contract’.

### Self-report measures

All measures were reported on a seven-point Likert scale (1 = strongly disagree, 7 = strongly agree) unless indicated otherwise, with the stem for each behaviour of ‘flossing my teeth in the evening’ and ‘taking a vitamin C tablet every day’.

#### Behaviour

At T0, participants reported baseline monthly frequency for both target behaviours. For flossing, participants were asked if they had ever flossed their teeth regularly before. At T1-T4, participants reported the number of times they had flossed in the evening, and had taken their vitamin C tablet in the past week. (Potential response options: 0–7 days.)

#### Habit

Habit was measured using the Self Report Behavioural Automaticity Index (SRBAI) [25], a reliable and valid subscale of the Self-Report Habit Index [26]. As the measurement was specifically of automaticity, the key component of habit, the habit concept will be indexed by “automaticity” throughout the Results section. For each behaviour, four items (e.g. ‘I do automatically’) followed the stem. (Combining all timepoints, flossing α = 0.98, vitamin C α = 0.98.) An option was added to the SRBAI (“N/A, I never floss my teeth in the evening/take vitamin C tablets”) to minimise misuse of the “neither agree nor disagree” response by participants who never perform the behaviour [27]. The “N/A” responses were assigned an automaticity score of zero, or treated as missing when there was a possibility of dormant habits (i.e., stored habit associations with the potential to elicit behaviour, but which rarely manifest in performance due to lack of exposure to associated cues) [28]. Dormant habits were deemed possible for participants who might have performed the target behaviour regularly before the intervention, which was determined from responses in the baseline questionnaires.^3^ Following the intervention, participants were judged to have been re-exposed to the cues, thus giving any dormant habits the opportunity to be manifested, so after the interventions, the “N/A, I never do behaviour X” response was assigned an automaticity score of zero.

#### Context stability

Participants were asked whether they perform the target behaviours ‘in the same place every time’ and ‘at the same point in my routine every time’ [29]. As in the SRBAI, an “N/A, I never…” option was included to minmise mid-point responding, and was treated as missing for participants with potential dormant habits. (Flossing α = 0.94, vitamin C α = 0.94.)

#### Intention

Intention was measured using two items: “I aim to...” and “I intend to...”. (Flossing α = 0.92, vitamin C α = 0.91.)**.**

#### Reward measures

For flossing, only the reward construct of pleasure was measured. For vitamin C, reward constructs were measured for: pleasure, intrinsic motivation, perceived utility and perceived benefits of the behaviour.

*Pleasure* measured how pleasant participants find the behaviour (e.g. is something I like a lot-dislike a lot). The *intrinsic motivation* measure was adapted from the exercise-specific BREQ-2 (Behavioural Regulation in Exercise) measure [30], and assessed identification (e.g. “…is important to me”), integration (e.g. “…is part of the way I have chosen to live my life”) and intrinsic motivation (e.g. “…is something I enjoy”). These were weighted as + 1, + 2 and + 3 respectively to calculate an overall score for autonomous motivation [30]. *Perceived utility* measured how generally useful participants think the behaviour is (e.g. very beneficial-very harmful). *Perceived benefits* (measured at T0, T1 and T4 only) measured the extent to which participants feel they could achieve specfic benefits from taking vitamin C tablets (e.g. reduction in length and severity of colds). This was measured using six items, on a 5-point Likert scale (I can definitely/definitely not achieve this). (For all constructs α > 0.79.) See Additional file 1: Appendix 1 for the full list of self-report measures.

### Statistical methods

Paired t-tests assessed whether the interventions had a significant effect on behaviour and automaticity, by comparing scores at the point of intervention administration (T0 for vitamin C, T1 for flossing) with scores at T4. (The participants assigned a missing initial automaticity score due to the potential for dormant habits could not be included in the t-test comparing pre-intervention with T4 automaticity, however they were included in all other analyses. Excluding those with potential dormant habits (i.e. those who may have simply reactivated dormant habits, as opposed to forming new habits,) from the comparison of initial and final habit scores, allowed for a conservative estimate of the effect of the intervention.) Structural Equation Modelling (SEM) was used to investigate dynamic predictors of habit formation, with separate models (comprising the same basic form) constructed at each timepoint. It was not possible to test a longitudinal model of such complexity, as the assumptions required would be too unrealistic (e.g. no unmeasured confounding across time periods, and having correct model specifications for all the additional time relationships between the variables). The difficulty of meeting assumptions would be exacerbated by the necessity of mediating pathways in order to test the second hypothesis. Therefore, it was deemed statistically appropriate to conduct separate models for each timepoint. Each reward construct was tested individually (i.e. without other reward variables present in the model), to assess the first hypothesis of whether each variable affects habit formation.

**Fig. 1:**
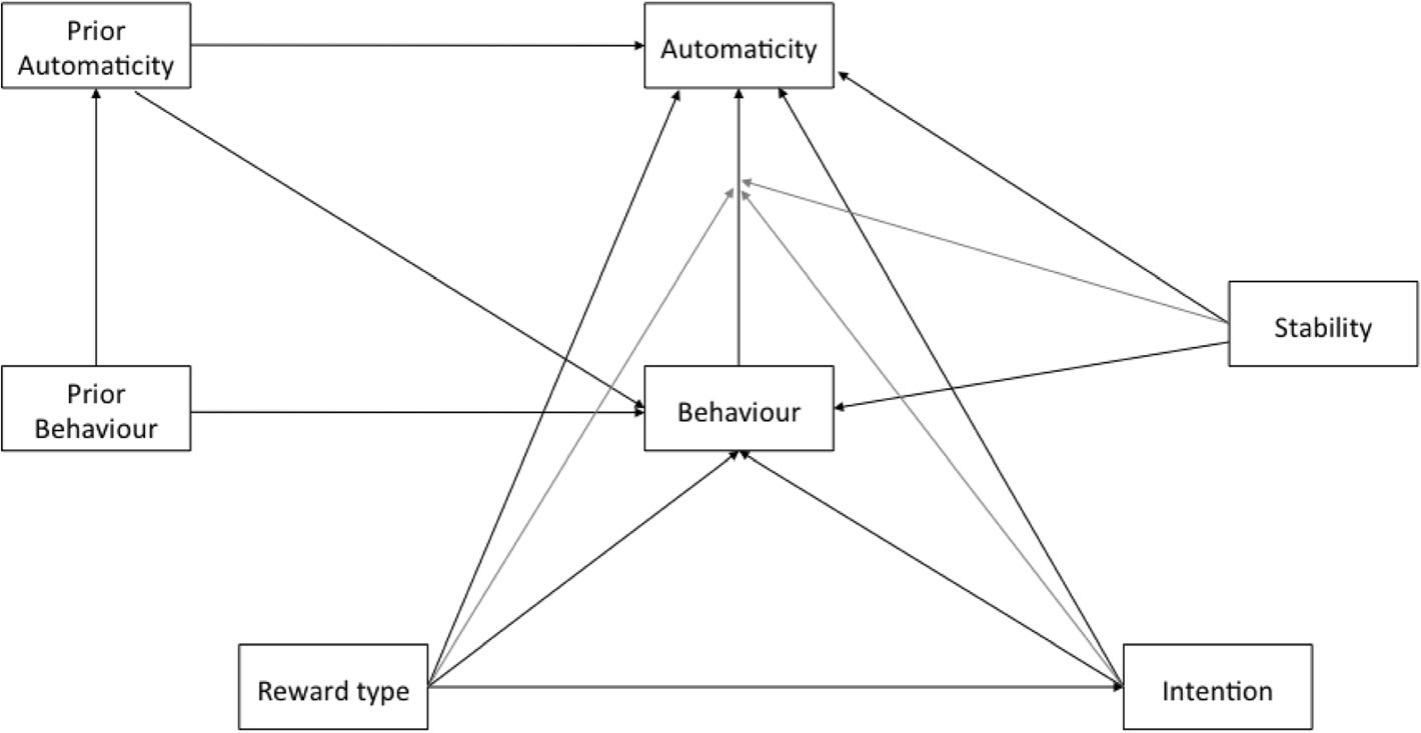
Full Structural Equation Model. Note. The term “Reward” here denotes each of the reward variables, which were tested in turn in separate models. The model was repeated for each of the timepoints T1-T4. Reward and behaviour, stability and behaviour, and intention and behaviour were allowed to interact in their effect on automaticity. This is termed a moderated effect, and is indicated on the diagram by the grey arrows. The reward, intention and context stability variables were those measured at the same timepoint as the behaviour and automaticity outcomes

The models were constructed to reflect known predictors of habit (as indexed by automaticity), and to address the hypothesis testing the mechanisms by which reward affects habit. The model is shown in Fig. 1. The basis of the model was that behaviour influences automaticity, and both automaticity and behaviour are influenced by their value at the previous timepoint. Another pathway allows behaviour to be influenced by automaticity at the previous timepoint. Pathways from reward to behaviour (and via intention to behaviour) were included to test for an effect of rewards on automaticity *mediated* by behaviour. An interaction term was created between reward and behaviour, and included as a predictor of automaticity, to test whether reward moderates the behaviour-automaticity relationship. A direct path from reward to automaticity was included for completeness, and to avoid an overly prescriptive model.

Intention and context stability were included, with paths to behaviour and automaticity. Interaction terms were created between behaviour and both intention and context stability, and included as predictors of automaticity. This controlling of theoretically expected covariates allows investigation of the mechanism of habit formation. The reward, intention and context stability variables were those from the same timepoint as the behaviour and automaticity outcomes, as intention should be measured close to the point of behaviour [31].

Behaviour data between T1 and T3 and context stability data was missing for 38 participants due to data collection problems, however, this was assumed to be missing at random (therefore not leading to bias in the results). Maximum Likelihood estimation was used to account for missing data. Each model’s goodness of fit was assessed using the Comparative Fit Index (CFI) and the Coefficient of Determination (CD, comparable to R-squared), with values close to one indicating a good fit.

## Results

### Baseline behaviour

At baseline, 26 participants (22%) reported that they had flossed regularly before, however current levels of flossing were very low. Thirty five (30%) participants reported flossing zero times per month on average, 43 (36%) reported flossing four times a month or less (i.e. not more than once a week), and only fourteen (12%) participants reported flossing more than eight times a month at baseline. For taking vitamin C tablets, 61 participants (76%) reported doing this zero times per month at baseline, thirteen participants (16%) reported taking vitamin C tablets not more than 4 times per month, and only five participants (6%) reported more than eight times per month.

### Intervention effect on behaviour and automaticity

Table 2 shows mean behaviour and automaticity scores for flossing and vitamin C from T0-T4. From T1 to T4, mean weekly flossing frequency increased significantly, t(88) = 8.29, *p* < .001 (Cohen’s d = 1.09), as did mean flossing automaticity, t(108) = 7.82, *p* < .001 (Cohen’s d = 0.82). Between T0 and T4, mean weekly vitamin C tablet consumption frequency increased significantly, t(77) = 5.30, *p* < .001 (Cohen’s d = 0.90), as did mean vitamin C automaticity, t(34) = 5.30, *p* < .001 (Cohen’s d = 1.03). The correlations between the different flossing variables, and the vitamin C variables are shown in Tables 3 and 4 respectively. Significant relationships were observed between all variables, except between perceived benefits and vitamin C behaviour at T4.

**Table 2:** *Mean behaviour frequency and automaticity for flossing and vitamin C throughout the study*

Outcome	Time 0 Mean (SD)	Time 1 Mean (SD)	Time 2 Mean (SD)	Time 3 Mean (SD)	Time 4 Mean (SD)	t test
Flossing behaviour	0.85 (1.01)	1.69 (1.91)	4.55 (2.42)	4.39 (2.41)	4.10 (2.58)	8.29***
Flossing automaticity	1.54 (1.55)	2.11 (1.73)	3.54 (1.81)	3.67 (1.96)	3.75 (2.07)	7.82***
Vitamin C behaviour	1.35 (2.60)	4.61 (2.59)	4.06 (2.80)	4.01 (2.88)	3.89 (2.14)	5.53***
Vitamin C automaticity	2.11 (1.25)	3.76 (1.90)	3.64 (2.10)	3.44 (2.13)	3.53 (2.31)	5.30***

Note: The behaviour values are recorded as weekly behaviour frequency

The t test results refer to the comparison between behaviour/automaticity levels at the point of the intervention and the end of the study. T0 values for vitamin C reflect pre-intervention levels, and T1 values for flossing reflect pre-intervention levels

*** *p* < .001

**Table 3:** Pairwise correlations between all flossing variables

	1.	2.	3.	4.
1. Behaviour^a^				
2. Automaticity^a^	.603***			
3. Pleasure	.465***	.678***		
4. Context stability	.470***	.438***	.421***	
5. Intention^b^	.518***	.532***	.592***	.366***

Note: ^a^Correlations with behaviour and automaticity are conducted at T4 only

^b^Skewed variable, and therefore all correlations with this variable are with Spearman’s rho

*** *p* < .001

**Table 4:** Pairwise correlations between all vitamin C reward variables

	1.	2.	3.	4.	5.	6.	7.
1. Behaviour^a^							
2. Automaticity^a^	.669***						
3. Pleasure	.344**	.621***					
4. Intrinsic motivation	.364**	.640***	.770***				
5. Perceived utility	.232*	.427***	.560***	.647***			
6. Perceived benefits	.150	.333**	.389***	.563***	.545***		
7. Context stability	.635***	.720***	.345***	.492***	.282***		
8. Intention^b^	.459***	.542***	.486***	.574***	.474***	.363***	.462***

Note: ^a^Correlations with behaviour and automaticity are conducted at T4 only

^b^ Skewed variable, and therefore all correlations with this variable are with Spearman’s rho

* *p* < .05, ** *p* < .01, *** *p* < .001

### Which reward variables impact upon automaticity gain and how?

To assess the hypotheses, Tables 5 and 6 display coefficients of the key pathways at each timepoint, from the reward variables to automaticity (i.e. mediated or moderated effects), for flossing and vitamin C respectively. (Complete SEM output is presented in Additional file 2: Appendix 2.) Pleasure and intrinsic motivation were positively related to automaticity via both the moderated and mediated pathways. Perceived utility and perceived benefits were not observed to be associated with automaticity gain. The relationships observed for each proposed reward variable are discussed below.

**Table 5:** Summary of reward relationships within the SEM models for flossing

Pathways from reward to automaticity	Time 1, b	Time 2, b	Time 3, b	Time 4, b
Mediated effect				
Reward-Behaviour	.248*	.417*	.127	−.010
			R-I: .530***	
			I-B: .316**	
Behaviour-Automaticity	.230	.266***	.260***	.174**
Moderated effect	−.010	.042	.112**	−.039

Note: for flossing, the intervention took place at T1 (at which point, so significant reward relationships were observed). For flossing, reward was measured in terms of pleasure

Due to complexity of the model, the mediated effect was not calculated, but instead, significant pathways along the mediated mechanism were reported. Significant mediated effects are only represented in the coefficients marked in bold, where more than one significant coefficient makes a complete significant pathway between reward to automaticity. (Items in italics reflect a mediation relationship from reward to automaticity that is via both via intention and behaviour, rather than just via behaviour. The coefficients from reward to intention are marked R-I, and the coefficients from intention to behaviour are marked I-B)

* *p* < .05, ** *p* < .01, *** *p* ≤ .001

**Table 6:** Summary of reward relationships within the SEM models for taking vitamin C tablets

Potential reward type	Pathway	Time 1, b	Time 2, b	Time 3, b	Time 4, b
Pleasure	Mediated				
	Reward-Behaviour	.215	.345*	−.066	−.116
	Behaviour Automaticity	.175*	.148*	.300***	.110
	Moderated	.048	.096*	.073*	.062
Intrinsic motivation	Mediated				
	Reward-Behaviour	.208	.120	.039	−.112
	Behaviour-Automaticity	.184*	.167**	.280***	.123
	Moderated	.066*	.056*	.028	.001
Perceived utility	Mediated				
	Reward-Behaviour	.323	−.177 ***R-I*** *:* .833*** I-B: .267**	.074	−.147
	Behaviour-Automaticity	.201**	.168**	.269***	.086
	Moderated	.086	.033	.059	.004
Perceived benefit	Mediated				
	Reward-Behaviour	−.410			−.234
	Behaviour-Automaticity	.205**	-	-	.088
	Moderated	.103			.037

Note: for vitamin C, the intervention took place at T0

Significant mediated effects are only represented in the coefficients marked in bold, where more than one significant coefficient makes a complete significant pathway between reward to automaticity. (Items in italics reflect a mediation relationship from reward to automaticity that is via both via intention and behaviour, rather than just via behaviour. The coefficients from reward to intention are marked R-I, and the coefficients from intention to behaviour are marked I-B). Perceived benefit was not measured at Time 2 or Time 3

* *p* < .05, ** *p* < .01, *** *p* ≤ .001

#### Pleasure: Flossing

Pleasure had a relationship with automaticity mediated by behaviour at T2, and mediated by intention and behaviour at T2 and T3. A moderation effect was observed at T3, whereby pleasure was associated with a stronger impact of behaviour on automaticity. Pleasure directly predicted T4 automaticity.

#### Pleasure: Vitamin C

Pleasure had a relationship with automaticity mediated by behaviour at T2. Pleasure was associated with a stronger behaviour-automaticity relationship at T2 and T3.

#### Intrinsic motivation: Vitamin C

Intrinsic motivation (not measured for flossing) had a moderated effect at T1 and T2, such that greater intrinsic motivation was associated with a stronger behaviour-automaticity relationship.

#### Perceived utility: Vitamin C

Perceived utility (not measured for flossing) only had the most indirect mediated relationship with automaticity, and only at T2, whereby perceived was associated with automaticity only via intention then behaviour.

#### Perceived benefit: Vitamin C

Perceived benefit (not measured for flossing) was not measured at T2 and T3. However, at T1 and T4 it was not related to automaticity.

### Effect of covariates on behaviour and automaticity

#### Intention

Flossing intention was significantly associated with behaviour at T2 and T3 only, respectively: *b* = .406, *p* = .021; *b* = .316, *p* = .007. For vitamin C, intention was only significantly associated with behaviour at T2, and only for the models including pleasure or perceived utility, respectively: *b* = .315, *p* = .047; *b* = .267, *p* = .006. For flossing, a moderation effect was seen whereby strong intentions were associated with a *weaker* impact of behaviour on automaticity at T3, *b* = −.082, *p* = .020. This negative moderation was also seen at T3 for the vitamin C models containing pleasure (*b* = −.092, *p* = .019), and intrinsic motivation (*b* = −.090, *p* = .036), where this effect was also found at T1 (*b* = −.116, *p* = .022).

However, for vitamin C, intention was directly associated with automaticity at T1 within the intrinsic motivation (*b* = .740, *p* = .013), perceived utility (*b* = .697, *p* = .023) and perceived benefits models (*b* = .598, *p* = .018). The findings indicate that while intention may be associated with higher automaticity, performing a behaviour more intentionally is likely to be associated with less automaticity *gain*.

#### Context stability

For flossing, context stability was significantly associated with behaviour at T1 and T2 (respectively: *b* = .298, *p* < .001; *b* = .436, *p* < .001). For vitamin C, context stability was significantly associated with behaviour at T1 for all models (plus at T2 in the perceived utility model, and T4 for the pleasure model) (within those six different models: *b* > .213, *p* < .046). Context stability did not moderate the flossing behaviour-automaticity relationship at any timepoint. Context stability was associated with a stronger vitamin C behaviour-automaticity relationship at T1 for all models, and T3 for the intrinsic motivation and perceived utility models (within those six different models: *b* > .062, *p* < .043).

Context stability was directly associated with flossing automaticity at T1 (*b* = .231, *p* = .001), and vitamin C automaticity at T4 for all models: pleasure (*b* = .224, *p* = .022), intrinsic motivation (*b* = .245, *p* = .014), perceived utility (*b* = .234, *p* = .021) and perceived benefits (*b* = .206, *p* = .042). This is at the point of the intervention for flossing (there is no corresponding model for vitamin C as the intervention was received at T0), and 16 weeks after the intervention for vitamin C (i.e. after the end of the study for flossing). Therefore context stability may predict automaticity when habits are not changing (i.e. before an intervention, or sufficiently after an intervention for automaticity to have stabilised).

For the flossing models, at T1 CD > .597 and CFI > .534, and from T2-T4 CD > .720 and CFI > .656. For the vitamin C models, at T1, CD > .537 and CFI > .334, and from T2-T4 CD > .714 and CFI > .601. This indicates acceptable model fit, though fit was poorer at T1, presumably due to weaker relationships between variables at baseline and post-intervention.

### Summary

Consistent with the first hypothesis, pleasure, and intrinsic motivation had an impact upon automaticity gain. However, contrary to the hypothesis, perceived utility and perceived benefits did not have an effect (perceived utility had a possible effect at one timepoint, mediated via both intention and behaviour). In support of the second hypothesis, both mediated and moderated effects of reward on automaticity were observed. However, the most consistent mechanism was the moderated effect, of reward being associated with a stronger impact of repetition on automaticity gain. This was most commonly observed eight weeks post-intervention (i.e. T3 for flossing, and T2 for vitamin C), as well as at adjacent timepoints.

At certain points, intention was associated with a weaker relationship between behaviour and automaticity gain for both flossing and vitamin C. Context stability was associated with flossing and vitamin C behaviour frequency. Higher levels of vitamin C context stability were also associated with a stronger behaviour-automaticity relationship.

## Discussion

This exploratory study investigated psychological variables that serve as rewards in habit formation, and the mechanisms by which they affect the habit formation process. Both behaviours increased in frequency following the intervention, as did habit strength, measured using automaticity as a proxy. Pleasure was associated with gains in flossing and vitamin C habit. Furthermore, intrinsic motivation was associated with increased vitamin C habit. Perceived utility or perceived benefits did not impact upon vitamin C behaviour or habit. While some rewarding variables had an effect on habit mediated by increased levels of behaviour repetition, the most consistent mechanism observed was the moderated effect, that finding the behaviour rewarding was associated with greater gains in habit per behavioural repetition. This is a novel finding, indicating that factors in addition to frequency of behavioural repetitions can affect the speed of habit formation. As this study is exploratory, practical implications that we discuss here are tentative, being dependent on the findings being robust. Replication studies, or follow up studies with a control condition, are needed to more rigorously test our hypotheses prior to guidelines being offered.

The factors found to be rewarding (through positive impact on habit formation), were those related to the experience of performing the behaviour (pleasure and intrinsic motivation), as opposed to anticipated outcomes of the behaviour (perceived utility and perceived benefits). This is consistent with accounts of affect and cognition as separate processes [32]. Just as positive sensory experience from food consumption leads to habit formation in animals [15], and dopamine is implicated in habit formation [33], it appears that reported pleasure, or positive sensory experiences in humans also predicts habit formation. The findings further support shared models of behaviour generation between humans and animals [13]; knowledge from animal habits research may be usefully applied to human habit formation. The results suggest that the efforts of manufacturers to make products more pleasurable to use may increase not only product use, but also habit. While it may be hard for health psychologists to manipulate the pleasure experienced from a behaviour, the field could gain from further consideration of how behaviours are experienced. It may be possible to increase reward value by drawing attention to positive outcomes that are typically less salient, or to develop strategies to reduce unpleasant aspects.

The mechanism observed most commonly (in all rewarding variables) was that higher levels of perceived rewards were associated with a stronger relationship between behavioural repetition and habit. This suggests that rewards may not solely operate by increasing the likelihood of behaviour repetition, but may also accelerate the formation of habits from a given number of repetitions. This moderation effect was largely seen eight weeks following the intervention. This timing may reflect a point when original intentions to perform a behaviour start to wane, and behaviour is more maintained by habitual processes, as has been observed in a study modelling habit formation [34]. Therefore there may be points during the habit formation process when certain effects are likely to occur. However, the observation of effects approximately eight weeks after the intervention may simply be a chance finding. Further, replication work is necessary to assess the robustness of our findings. Findings are consistent with theories of reinforcement learning [13], whereby reward can positively reinforce a behaviour by strengthening the connection between a stimulus (e.g. the context) and the response. The more rewarding a behaviour, the greater the reinforcement, resulting in greater gains in habit for a given frequency of behaviour. This moderation of the behaviour-habit relationship by intrinsic motivation has previously been observed in studies of pre-existing habits [6, 7], but the present study demonstrates this effect in the process of forming new habits. Investigating the formation of new habits allows inferences about the habit formation process to be made more reliably, as opposed to documenting between-person variation in existing habits and relationships between habit strength and potential correlates. The results suggest that interventions can be designed to lead to stronger habits from a given number of repetitions, before intentions may wane [34], thus promoting more sustained healthy behaviour change.

As intrinsic motivation strengthened the behaviour-habit relationship, this may further explain why intrinsic motivation is established as a more effective means to sustained behaviour change than extrinsic motivation [35, 36], and why financial incentives (i.e. extrinsic rewards) do not have a long-term impact on regularly repeated behaviours such as smoking and exercise [37]. That intrinsic motivation promoted habit formation suggests that interventions could be made more effective by targeting individuals who are intrinsically motivated, encouraging people to make self-directed changes in behaviour, or proactively fostering intrinsically motivated behaviour. According to Self-Determination Theory, intrinsic motivation can be encouraged by fostering autonomy, competence, and connection with others [16, 36], e.g. using strategies such as self-monitoring of performance, and positive feedback [35]. Intrinsic motivation can also be encouraged through support by people to whom the participant can relate [36], e.g. non-professionals with personal experiences related to the target behaviour [38].

Longer-term cognitions about the perceived usefulness of a behaviour did not have a positive impact on behaviour or habit except through intention. This is consistent with reviews finding that instrumental attitude has an impact upon intention, but not behaviour [39]. Furthermore, it may be that performing a behaviour with an outcome in mind, strengthens the perceived contingency between the behaviour and outcome (reward), resulting in behaviour remaining goal-directed rather than becoming habitual [10, 40]. This contrasts with previous research where attitude predicted flossing habit after four weeks [11], but is consistent with findings from a review of physical activity interventions, which found that interventions focussing on consequences of behaviour were less effective at sustaining physical activity after twelve months [41].

There was limited impact of intention on behaviour, particularly for vitamin C adherence, reflecting the literature that intentions are only weakly predictive of behaviour, particularly for regularly performed behaviours [42, 43]. Stronger intentions were commonly associated with smaller increases in automaticity, potentially as strong intentions increase the salience of behavioural goals, and greater perceived contingency between the behaviour and outcomes impedes habit formation [44].

Habits form by ‘context-dependent repetition’ or repeated pairings of performance contexts and behaviour [5, 9]. However, the mechanism by which the stability of context influences habit formation has not been tested. In the present study, performing a behaviour in a more stable context (measured here as location and point within a routine) was associated with more frequent repetition, thus supporting habit formation. This is likely due to salient aspects of the context becoming more effective reminders to perform the behaviour if they are stable and therefore more uniquely associated with the behaviour.

Taking vitamin C tablets in a more stable context was associated with greater gains in habit per behavioural repetition. This may be expected, as if a behaviour is performed in a stable context, each repetition would lead to greater automaticity gain due to strengthening of associations between the context and behaviour. If instead the behaviour is performed in different situations, it would be harder to associate cues with the behaviour, and so context dependent automaticity would be less likely [9]. Yet this effect was not found for flossing. Direct effects of context stability were also observed, however only at points when habit strength was not changing, suggesting that stability is related to habit strength only for stable habits [4].

One unexpected observation was that after an initial increase, rather than plateauing [5], scores subsequently decreased for both behaviours and for vitamin C automaticity (though these decreases were not significant). A potential explanation of this finding may draw on work modelling the habit formation process by Tobias [34]. Following an intervention, motivation is high, and this is what sustains behaviour. However, over time, motivation and memory for the new behaviour decline. This leads to decreases in behaviour frequency, unless habits have formed. Therefore, it may be possible that mean scores for behaviour following an intervention increase and then gradual decreases are observed. Likewise, while habit scores may increase following 4 weeks of initial performance, if those newly formed habits are not yet strong enough to consistently sustain the behaviour while motivation and memory decline, the habit scores may also decrease over time due to the declining behavioural frequency.

Limitations of this study must be acknowledged. Participants received interventions for two behaviours, so there may have been interference due to attempts to form different habits within the same study. However, the separation of four weeks between the two interventions meant that the initiation period of the two behaviours would not overlap, and throughout the study, the two behaviours would have been at different stages in the habit formation process. Another investigation into a habit formation intervention found that habit gains were of similar magnitude for goals pursued either individually, or alongside other goals [45]. Also, as the study investigated the effect of perceived rewards on habit formation, as opposed to testing the efficacy of a particular intervention, the monitoring of two behaviours would be unlikely to affect the findings. While we used an intervention design to investigate habit formation, we did not manipulate the different potentially rewarding variables, limiting the extent to which causality can be inferred. Furthermore, the baseline rates of the target behaviours were in some cases relatively high, such that some participants were increasing the frequency of a behaviour, rather than initiating a novel behaviour. We do not know whether some participants were rediscovering old habits, adding the behaviour to a related pre-existing habit (e.g. adding vitamin C tablets to a pre-existing medication habit), bolstering weak habits, or forming entirely new habits.

Other limitations of the study include reliance on self-report measures. Self-report of behaviour frequency can be vulnerable to bias and memory failure. While the study was initially designed to use electronic sensors to objectively monitor behaviour, these were unreliable, and so the data could not be used for analysis. Self-report habit measures have also been criticised, due to reliance on people’s reports on their subconscious action [46], and as they measure perceptions of habit, as opposed to the underlying habituation associations. However, while people may be unaware of the strength of automatic processes when they generate behaviour, they may still be aware of the development of automaticity [47], and it can be impractical to use more ‘objective’ reaction time measures outside the lab. Nonetheless, there has not been sufficient testing of the reliability of self-report habit measures, or their ability to monitor change in habits over time. Further research is needed to investigate the validity of self-report measures of habit for measuring change in habit over time, and to develop more objective measures of habit that are easier to administer outside of a lab setting.

## Conclusions

This study is the first to investigate the impacts of perceived rewards on the mechanism of habit formation. Behaviours that are pleasurable or intrinsically motivating, may become habitual after fewer repetitions than those that are not, as pleasure and intrinsic motivation act as rewards, which accelerate habit formation. Perceived utility or benefits of vitamin C adherence did not have this effect, suggesting that experiences of performing a behaviour have a greater impact on habit formation than cognitions around performing the behaviour, which do not serve as rewards. Reward strengthened the repetition-habit gain relationship, demonstrating that the impact of the rewarding variables on habit was not merely mediated by higher levels of behaviour. In addition, performing a behaviour in a stable context led to more frequent behaviour, but may also strengthen the effect of each repetition on habit formation. These findings should be applied to the design of behaviour change interventions so as to accelerate the habit formation process.

## Additional files

Additional file 1: **Appendix 1** full list of self-report measures. (DOCX 27.7 KB)

Additional file 2: **Appendix 2** full SEM output. (DOCX 52 kb)

## Abbreviations

BREQ-2: Behavioural regulation in exercise questionnaire-2
CD: Coefficient of determination
CFI: Comparative fit index
SEM: Structural equation modelling
SRBAI: Self-report behavioural automaticity index

## Footnotes

1 Only 80 of the 118 participants received the vitamin C intervention, as initially one of the interventions was for handwashing with soap after using the toilet. However, after 38 participants were recruited, baseline automaticity was observed to be high, resulting in limited scope to change behaviour or automaticity. Therefore, the second target behaviour was changed to taking vitamin C tablets for the remaining participants. Two behaviours were investigated to maximise the output due to high study costs, as the research used electronic sensors to monitor behaviour. However, due to numerous problems with the sensors, the data was not sufficiently reliable to report here.

2 The flossing intervention included a two-by-two design: participants were randomised to floss before or after brushing (Judah et al., 2013), and to an intervention including consequences based on health benefits or physical attractiveness. However, behaviour frequency or habit formation did not differ between conditions, so these aspects are not discussed further here. Therefore, the analyses presented here for flossing are of available data from a study that was not designed to primarily test our current hypotheses. Results from the different conditions were combined for this analysis. None of the results described below differed when analyses were rerun to control for intervention condition.

3 For flossing, participants were asked at baseline whether they had ever flossed regularly before, so participants who answered yes to this question who also gave the “N/A, I never floss my teeth” response to the SRBAI were considered to potentially have dormant habits. For vitamin C, there was not a comparable question about past regular behaviour, with participants just asked at baseline the number of times they take vitamin C tablets per month on average. Therefore, all participants who responded “N/A, I never take vitamin C tablets” may have potentially had dormant habits given the data available.
