# Supplementary material for: Exploratory study of the impact of perceived reward on habit formation
Source: BMC Psychol. 2018 Dec 20;6:62. doi: 10.1186/s40359-018-0270-z (PMC6302524; doi:10.1186/s40359-018-0270-z)
Supplement: Supplementary file 1 — Appendix 1 full list of self-report measures. (DOCX 27.7 KB) [file 40359_2018_270_MOESM1_ESM.docx]

**Additional file 1: Full list of self-report measures**

For flossing, the reward construct of pleasure was measured. For vitamin C, reward constructs were measured for: pleasure, intrinsic motivation, perceived utility and perceived benefits of the behaviour. More constructs were tested for vitamin C compared to flossing as the initial 2x2 design for the flossing intervention meant fewer reward constructs could be tested.

The items for each construct are shown in Tables 1 and 2. There are slight differences in the items between the two behaviors, due to the later introduction of vitamin C, along with more reward constructs.

The intrinsic motivation scores were calculated from weighted scores from the constructs measuring identification, integration and intrinsic motivation, from a scale of autonomous motivation ([Markland & Tobin, 2004](#_ENREF_189)). (These were weighted as +1, +2 and +3 respectively). Identification represents a conscious acceptance of the behaviour as being important in order to achieve personally valued outcomes. Integrated motivation concerns the assimilation of identified regulation so that performing the behaviour is fully consistent with one’s sense of self. Intrinsic motivation involves performing an activity for the enjoyment and satisfaction inherent in engaging in the behaviour itself. While introjected and external motivation and amotivation were also measured, they did now show the expected patterns of correlations with the other items, so the overall score for intrinsic motivation excluded those constructs on the extrinsic side of the spectrum.

| Construct | Items | Cronbach’s alpha |
| --- | --- | --- |
| Behaviour | How many evenings in the past week have you flossed your teeth? (Responses 0-7) | n/a |
| Habit | Flossing my teeth in the evening is something…  1) …I do without having to consciously remember  2) …I do automatically  3) …I do wihout thinking  4) …I start doing before I realise I am doing it | 0.98 |
| Context stability | When I floss my teeth I do it in the same place every time  When I floss my teeth I do it in the same point in my routine every time | 0.94 |
| Intention | I aim to floss my teeth every evening  I intend to floss my teeth every evening | 0.92 |
| Pleasure | I like flossing my teeth every evening  I think flossing every evening:  Is very enjoyable-very unenjoyable  Is very pleasurable-very unpleasurable  feels pleasant to do | 0.92 |

Table 1 Self-report measures for flossing

All items were measured on a seven-point Likert scale (1=Strongly disagree, 7=Strongly agree), unless indicated otherwise. The mean of the items was calculated for the overall item score.

| Construct | Items | Cronbach’s alpha |
| --- | --- | --- |
| Behaviour | In the past week, on how many days have you taken your vitamin C tablet? (Responses 0-7) | n/a |
| Habit | Taking a vitamin C tablet every day is something…  1) …I do without having to consciously remember  2) …I do automatically  3) …I do wihout thinking  4) …I start doing before I realise I am doing it | 0.98 |
| Context stability | When I take a vitamin C tablet I do it in the same place every time  When I take a vitamin C tablet I do it in the same point in my routine every time | 0.94 |
| Intention | I aim to take a vitamin C tablet every day  I intend to take a vitmain C tablet every day | 0.91 |
| Pleasure | Taking a vitamin C tablet is:  Pleasant-unpleasant  Something I like a lot-something I dislike a lot | 0.82 |
| Perceived utlity | Taking a vitamin C tablet every day is:  Very beneficial-very harmful  Very useful-very useless | 0.85 |
| Perceived benefits (measured at T0, T1 and T4 only) | To what extent do you believe that if you took vitamin C tablets, you would achieve the following benefits:  - reduction in the length and severity of colds  - reduction in effects of aging on skin  - protection against cancer and cardiovascular disease  - protection against damage caused by exercise  - protection of the immune system against stress  - protection against the effects of carbon monoxide on vitamin levels  (Responses on a five-point Likert scale: 1=I would definitely not achieve this, 5=I would definitely achieve this) | 0.91 |
| Relative autonomy subscales (overall Cronbach’s alpha 0.91) | | |
| Identification | Taking a vitamin C tablet is good for my health  Taking a vitamin C tablet is important to me  I value the benefits of taking a vitamin C tablet | 0.81 |
| Integration | Taking a vitamin C tablet is consistent with my values  Taking a vitamin C tablet is an integral part of my life  Taking a vitamin C tablet is part of the way I have chosen to live my life | 0.83 |
| Intrinsic motivation | Taking a vitamin C tablet is something I like doing  Taking a vitamin C tablet gives me pleasure and satisfaction  Taking a vitamin C tablet is something I enjoy | 0.93 |

Table 2 Self-report measures for taking a vitamin C tablet

All items were measured on a seven-point Likert scale (1=Strongly disagree, 7=Strongly agree), unless indicated otherwise. The mean of the items was calculated for the overall item score.

Note: for the baseline questionnaire when people do not necessarily have experience of taking vitamins, the questions were reworded in the form: if I were to take vitamins during the study period, it would be because...
