# Supplementary material for: Exploratory study of the impact of perceived reward on habit formation
Source: BMC Psychol. 2018 Dec 20;6:62. doi: 10.1186/s40359-018-0270-z (PMC6302524; doi:10.1186/s40359-018-0270-z)
Supplement: Supplementary file 2 — Appendix 2 full SEM output. (DOCX 52 kb) [file 40359_2018_270_MOESM2_ESM.docx]

**Additional file 2: Full tables of Structural Equation Modelling output**

| Pleasure | T1, N=118 | T2, N=118 | | T3, N=117 | | | T4, N=115 |
| --- | --- | --- | --- | --- | --- | --- | --- |
| Hypothesised predictors of automaticity | | |  | | |  | |
| Past automaticity | .303*** | .141 | | .414*** | | | .508*** |
| Behaviour | **.230** | **.266***** | | **.260***** | | | **.174**** |
| Intention | .027 | .132 | | .191 | | | -.249 |
| Stability | .231*** | .140 | | .053 | | | .157 |
| Pleasure | .197 | .381 | | -.008 | | | .461* |
| Intention * Behaviour Interaction | -.036 | -.046 | | -.082* | | | .077 |
| Stability * Behaviour Interaction | -.035 | -.024 | | -.032 | | | -.042 |
| Pleasure * Behaviour Interaction | **-.010** | **.042** | | **.112**** | | | **-.039** |
| Hypothesised predictors of behaviour | | | | | |  | |
| Past automaticity | -.038 | -.223 | | -.005 | | | .101 |
| Intention | -.160 | .406* | | .316** | | | .164 |
| Stability | .298*** | .436*** | | .131 | | | .081 |
| Pleasure | **.248*** | **.417*** | | **.127** | | | **-.010** |
| Past behaviour | .073 | .303*** | | .648*** | | | .666*** |
| Hypothesised predictors of past automaticity | | | | | | |  |
| Past behaviour | .145*** | .465*** | | .477*** | | | .525*** |
| Hypothesised predictor of intention | | | | |  | | |
| Pleasure | .398*** | .600*** | | .530*** | | | .628*** |
|  |  |  | |  | | |  |
| CD | .636 | .723 | | .864 | | | .829 |
| CFI | .588 | .719 | | .743 | | | .656 |

*Table 1 Flossing SEM results with Pleasure as a rewarding variable*

** p* *<.05, ** p<.01, *** p≤.001*

All predictors are measured at the same timepoint as the outcome measurement of behaviour and automaticity (i.e. T). The predictors labelled “past automaticity” and “past behaviour” are measured at the previous timepoint (i.e. T-1).

*Key coefficients indicating the moderated and mediated pathways are marked in bold.*

The goodness of fit is assessed using the Comparative Fit Index (CFI) and the Coefficient of Determination (CD), for both of which, values close to one indicate a good fit.

| Pleasure | T1, N=80 | T2, N=79 | | T3, N=79 | | | T4, N=79 |  |  |  |
| --- | --- | --- | --- | --- | --- | --- | --- | --- | --- | --- |
| Hypothesised predictors of automaticity | | |  | | |  | |  |  |  |
| Past automaticity | .128 | .511*** | | .458*** | | | .440*** |  |  |  |
| Behaviour | **.175*** | **.148**** | | **.300***** | | | **.110** |  |  |  |
| Intention | .465 | .121 | | .260 | | | .205 |  |  |  |
| Stability | .123 | .177 | | -.039 | | | .224* |  |  |  |
| Pleasure | .178 | .060 | | -.112 | | | .091 |  |  |  |
| Intention * Behaviour Interaction | -.076 | -.027 | | -.092* | | | -.033 |  |  |  |
| Stability * Behaviour Interaction | .097* | .015 | | .048 | | | .009 |  |  |  |
| Pleasure * Behaviour Interaction | **.048** | **.096*** | | **.073*** | | | **.062** |  |  |  |
| Hypothesised predictors of behaviour | | | | | |  | |  |  |  |
| Past automaticity | -.070 | -.298* | | .064 | | | .253 |  |  |  |
| Intention | .100 | .315* | | .367 | | | .018 |  |  |  |
| Stability | .902*** | .248 | | .254 | | | .213* |  |  |  |
| Pleasure | **.215** | **.345*** | | **-.066** | | | **-.116** |  |  |  |
| Past behaviour | .061 | .679*** | | .480*** | | | .530*** |  |  |  |
| Hypothesised predictors of past automaticity | | | | | | |  |  |  |  |
| Past behaviour | **-.018** | **431***** | | **.428***** | | | **.525***** |  |  |  |
| Hypothesised predictor of intention | | | | |  | | |  |  |  |
| Pleasure | .424*** | .557*** | | .517*** | | | .676*** |  |  |  |
|  |  |  | |  | | |  |  |  |  |
| CD | .607 | .819 | | .730 | | | .841 |  |  |  |
| CFI | .394 | .675 | | .601 | | | .653 |  |  |  |

*Table 2 Vitamin SEM results with Pleasure as a rewarding variable*

** p* *<.05, ** p<.01, *** p≤.001*

All predictors are measured at the same timepoint as the outcome measurement of behaviour and automaticity (i.e. T). The predictors labelled “past automaticity” and “past behaviour” are measured at the previous timepoint (i.e. T-1).

*Key coefficients indicating the moderated and mediated pathways are marked in bold.*

The goodness of fit is assessed using the Comparative Fit Index (CFI) and the Coefficient of Determination (CD), for both of which, values close to one indicate a good fit.

| Intrinsic motivation | T1, N=80 | T2, N=79 | | T3, N=79 | | | T4, N=79 |  |  |  |
| --- | --- | --- | --- | --- | --- | --- | --- | --- | --- | --- |
| Hypothesised predictors of automaticity | | |  | | |  | |  |  |  |
| Past automaticity | .170 | .492*** | | .469*** | | | .433*** |  |  |  |
| Behaviour | **.184*** | **.167**** | | **.280***** | | | **.123** |  |  |  |
| Intention | .737* | .034 | | .234 | | | -.108 |  |  |  |
| Stability | .048 | .185 | | -.050 | | | .245* |  |  |  |
| Intrinsic motivation | -.181 | .078 | | -.008 | | | .246 |  |  |  |
| Intention * Behaviour Interaction | -.116* | -.039 | | -.090* | | | -.002 |  |  |  |
| Stability * Behaviour Interaction | .101* | .016 | | .060* | | | .016 |  |  |  |
| Intrinsic motivation * Behaviour Interaction | **.066*** | **.056*** | | **.028** | | | **.001** |  |  |  |
| Hypothesised predictors of behaviour | | | | | |  | |  |  |  |
| Past automaticity | -.070 | -.285* | | .024 | | | .276 |  |  |  |
| Intention | .047 | .303 | | .323 | | | .106 |  |  |  |
| Stability | .884*** | .259 | | .256 | | | .199 |  |  |  |
| Intrinsic motivation | **.208** | **.120** | | **.039** | | | **-.112** |  |  |  |
| Past behaviour | .036 | .688*** | | .490*** | | | .523*** |  |  |  |
| Hypothesised predictors of past automaticity | | | | | | |  |  |  |  |
| Past behaviour | -.018 | .432*** | | .428*** | | | .525*** |  |  |  |
| Hypothesised predictor of intention | | | | |  | | |  |  |  |
| Intrinsic motivation | .278*** | .411*** | | .384*** | | | .493*** |  |  |  |
|  |  |  | |  | | |  |  |  |  |
| CD | .685 | .867 | | .778 | | | .909 |  |  |  |
| CFI | .377 | .714 | | .608 | | | .637 |  |  |  |

*Table 3 Vitamin SEM results with intrinsic motivation as a rewarding variable*

** p* *<.05, ** p<.01, *** p≤.001*

All predictors are measured at the same timepoint as the outcome measurement of behaviour and automaticity (i.e. T). The predictors labelled “past automaticity” and “past behaviour” are measured at the previous timepoint (i.e. T-1).

*Key coefficients indicating the moderated and mediated pathways are marked in bold.*

The goodness of fit is assessed using the Comparative Fit Index (CFI) and the Coefficient of Determination (CD), for both of which, values close to one indicate a good fit.

| Perceived utility | T1, N=80 | T2, N=79 | | T3, N=79 | | | T4, N=79 |  |  |  |
| --- | --- | --- | --- | --- | --- | --- | --- | --- | --- | --- |
| Hypothesised predictors of automaticity | | |  | | |  | |  |  |  |
| Past automaticity | .167 | .615*** | | .534*** | | | .549*** |  |  |  |
| Behaviour | **.201**** | **.168**** | | **.269***** | | | **.086** |  |  |  |
| Intention | .697* | .011 | | .251 | | | .021 |  |  |  |
| Stability | .013 | .132 | | -.075 | | | .234* |  |  |  |
| Perceived utility | -.360 | .336 | | -.161 | | | .349 |  |  |  |
| Intention * Behaviour Interaction | -.098 | .001 | | -.077 | | | .002 |  |  |  |
| Stability * Behaviour Interaction | .107* | .013 | | .062* | | | .016 |  |  |  |
| Perceived utility * Behaviour Interaction | **.086** | **.033** | | **.059** | | | **.004** |  |  |  |
| Hypothesised predictors of behaviour | | | | | |  | |  |  |  |
| Past automaticity | -.028 | -.245 | | .038 | | | .218 |  |  |  |
| Intention | .099 | .267** | | .332 | | | .035 |  |  |  |
| Stability | .907*** | .275* | | .247 | | | .206 |  |  |  |
| Perceived utility | **.323** | **-.177** | | **.074** | | | **-.147** |  |  |  |
| Past behaviour | .064 | .663*** | | .493*** | | | .544*** |  |  |  |
| Hypothesised predictors of past automaticity | | | | | | |  |  |  |  |
| Past behaviour | -.015 | .432*** | | 428*** | | | .525*** |  |  |  |
| Hypothesised predictor of intention | | | | |  | | |  |  |  |
| Perceived utility | .471** | .833*** | | .638*** | | | .882*** |  |  |  |
|  |  |  | |  | | |  |  |  |  |
| CD | .593 | .791 | | .721 | | | .842 |  |  |  |
| CFI | .334 | .681 | | .616 | | | .652 |  |  |  |

*Table 4 Vitamin SEM results with Perceived utility as a rewarding variable*

** p* *<.05, ** p<.01, *** p≤.001*

All predictors are measured at the same timepoint as the outcome measurement of behaviour and automaticity (i.e. T). The predictors labelled “past automaticity” and “past behaviour” are measured at the previous timepoint (i.e. T-1).

*Key coefficients indicating the moderated and mediated pathways are marked in bold.*

The goodness of fit is assessed using the Comparative Fit Index (CFI) and the Coefficient of Determination (CD), for both of which, values close to one indicate a good fit.

| Perceived benefits | T1, N=80 | T4, N=79 | | |  |  |  |
| --- | --- | --- | --- | --- | --- | --- | --- |
| Hypothesised predictors of automaticity | | |  | |  |  |  |
| Past automaticity | .193 | .548*** | | |  |  |  |
| Behaviour | **.205**** | **.088** | | |  |  |  |
| Intention | .598* | .143 | | |  |  |  |
| Stability | .042 | .206* | | |  |  |  |
| Perceived benefits | -.290 | .145 | | |  |  |  |
| Intention * Behaviour Interaction | -.086 | -.019 | | |  |  |  |
| Stability * Behaviour Interaction | .107* | .025 | | |  |  |  |
| Perceived benefits * Behaviour Interaction | **.103** | **.037** | | |  |  |  |
| Hypothesised predictors of behaviour | | | |  |  |  |  |
| Past automaticity | -.032 | .217 | | |  |  |  |
| Intention | .203 | .052 | | |  |  |  |
| Stability | .862*** | .207 | | |  |  |  |
| Perceived benefits | **-.410** | **-.234** | | |  |  |  |
| Past behaviour | .111 | .537*** | | |  |  |  |
| Hypothesised predictors of past automaticity | | | | |  |  |  |
| Past behaviour | -.018 | .525*** | | |  |  |  |
| Hypothesised predictor of intention | | | | |  |  |  |
| Perceived benefits | .359 | 1.124*** | | |  |  |  |
|  |  |  | | |  |  |  |
| CD | .537 | .830 | | |  |  |  |
| CFI | .369 | .671 | | |  |  |  |

*Table 5 Vitamin SEM results with Perceived benefits as a rewarding variable*

** p* *<.05, ** p<.01, *** p≤.001*

All predictors are measured at the same timepoint as the outcome measurement of behaviour and automaticity (i.e. T). The predictors labelled “past automaticity” and “past behaviour” are measured at the previous timepoint (i.e. T-1).

*Key coefficients indicating the moderated and mediated pathways are marked in bold.*

The goodness of fit is assessed using the Comparative Fit Index (CFI) and the Coefficient of Determination (CD), for both of which, values close to one indicate a good fit.
